# A Novel Deep Framework for English Communication Based on Educational Psychology Perspective

**DOI:** 10.3389/fpubh.2022.916101

**Published:** 2022-06-21

**Authors:** Ying Wang, Liang Zheng

**Affiliations:** ^1^School of Foreign Studies, North China University of Water Resources and Electric Power, Zhengzhou, China; ^2^School of Foreign Languages, Henan University of Engineering, Zhengzhou, China

**Keywords:** cognitive psychology, English communication, oral English, psychological model, strategy formulation

## Abstract

The impact of verbal reading practices on learning is examined from the perspective of educational psychology, using the motivation theory and the schema theory. This research intends to enhance learner's English communication abilities in response to the needs for national economic growth and scientific and technological development. To motivate students to improve their English, the research may address the issue of inadequate opportunities by adding an artificial intelligence (AI) conversation mechanism to the students speaking English exercise. First, cognitive psychology is analyzed in detail, and a model based on cognitive psychology is implemented to solve the problems existing in student's English communication. In addition, various measures are presented and used to increase student's oral English communication abilities. We used sixty students from North China University of Water Resources and Electric Power are separated into two classes: Class A and Class B. The experimental group is called Class A, while the control group is called Class B. Following a comparison of the outcomes obtained before and after training. The experimental group's reading comprehension, responding to questions, situational conversation, and subject description scores rose by 13.33, 15.19, 17.39, and 28.3 %, respectively. The overall average score of the class climbed by 17.75 %, whereas the scores of pupils in Class B improved just an undersized. The results reveal that following the vocalized reading exercise, the student's English grades, self-efficacy, and topic knowledge increased considerably in the experimental group. Moreover, the proposed model, employs computer simulation in the English communication teaching system and AI, which can aid in the creation of an interactive learning environment for students to improve their spoken English and English communication abilities.

## Introduction

The study of artificial neural networks is a significant topic in the fields of English communication and psychology technology. It is also the fundamental rudiment of deep learning neural network structure. With social globalization, foreign language has gradually become an indispensable tool for communicating with the outside world and other countries. English is the most widely used language in global communication as a universal language. More than 2 billion people around the globe often communicate in English. Forty-five countries take English as their official language. Thus, English has become an essential tool for international, scientific, technological, and cultural exchanges and a skill for knowing about the achievements of human civilization and advanced technology. English ability has also become the core competence of talent competition as an element to promote the integration of Chinese and foreign cultures (1).

School instructors, on the other hand, normally place a greater emphasis on enhancing pupils' reading and writing abilities. Few people pay attention to their oral English communication, resulting in many students' incapacity to speak effectively after more than 10 years of study. In this instance, English instruction should not be focused on passing English examinations, but rather on developing talent for national economic development and scientific and technical advancement. Students' capacity to get information, process it, solve issues, and communicate themselves in English should also be improved (2).

The English language is one of the most widely used languages in scientific study and collaboration. Each country has its unique qualities when it comes to the application of English grammar. There are apparent changes in English grammar, for example. In the same nation, there are also grammatical and semantic variances across states and cities. A fundamental technique in simultaneous translation and English writing is the ability to intelligently recognize and evaluate English grammar. By reducing ambiguity in English grammar and semantics, we may increase the efficiency of English reading, communication, and communication. The semantic character and recognition impact will be influenced during the real detection procedure. Researchers have built comparable network topologies as part of their study of neural network structure analysis and mechanism. It may be used to provide a big amount of learning data to the computer model during the training phase for legislation interpretation. As a result of the creation of the inversion method, artificial neural network research has reached a fever pitch. Theoretical analysis is challenging due to a lack of a thorough grasp of the algorithm and network topology. The neural network topology during this time is quite basic, and the majority of its internal structure is made up of single-level neurons, which are mostly used for shallow learning and training studies.

In this study, we proposed a cognitive psychology framework and a paradigm for English communication. A deep neural network algorithm and prediction model were used in this paper for the study and exploration of English grammar and communication. English communication plays an increasingly essential role in the fields of speech, vocabulary, text, pictures, and other information processing studies. Based on this, various solutions for improving students' English communication skills are created and implemented in response to the challenges in students' English communication. The findings before and after exercise are compared between the experimental and control groups after 2 months of training, indicating that the model and recommended tactics are scientific and practicable. The results suggest that an English grammar detection system based on deep learning enhances overall detection accuracy by simplifying the process structure in the recognition process, reducing superfluous operation cycles, simplifying the process structure in the recognition process, and helping students improve their English communication skills.

## Materials and Methods

### Dataset Processing

In order to meet the demand for cognitive semantic analysis in English grammar detection, we constructed 500 sentences by randomly picking phrase structure rules. If the language allowed for single-clause or dual-clause sentences, we would choose a single-clause sentence at random. The stroll went down to the noun phrase structural rule because single-clause sentences needed a noun phrase subject. When given the choice of a singular or plural subject noun phrase, we would choose the plural noun phrase at random, and when given the choice of an adjective modifier or not, we would choose no adjective at random. This procedure was followed for each of the sentence's syntactic components, right down to the word choice.

### Psychology Model Analysis

Cognitive psychology is born in the mid-1950s. It is a discipline with the mental process in human psychological phenomena as the primary research object. It is an essential branch of cognitive science in a broad sense (including computer science, communication science, linguistics, logic, and anthropology) (3, 4). It falls into broad psychology and narrow psychology. Generally, broad psychology includes constructivism psychology, psychologism, and information-processing psychology. Constructivist Psychology holds the idea that the research object of psychology is conscious experience. Psychology should use experimental introspection to analyze the content or structure of consciousness and find out the components of consciousness and the laws of their complex psychological process. Psychologism depicts human and animal psychological processes and is mostly used to identify activities through observation. Information processing psychology is the study of human cognition through the comparison of cognitive functions to computer processing systems. In a narrow sense, cognitive psychology is also information processing psychology (5–8).

**Figure 1:**
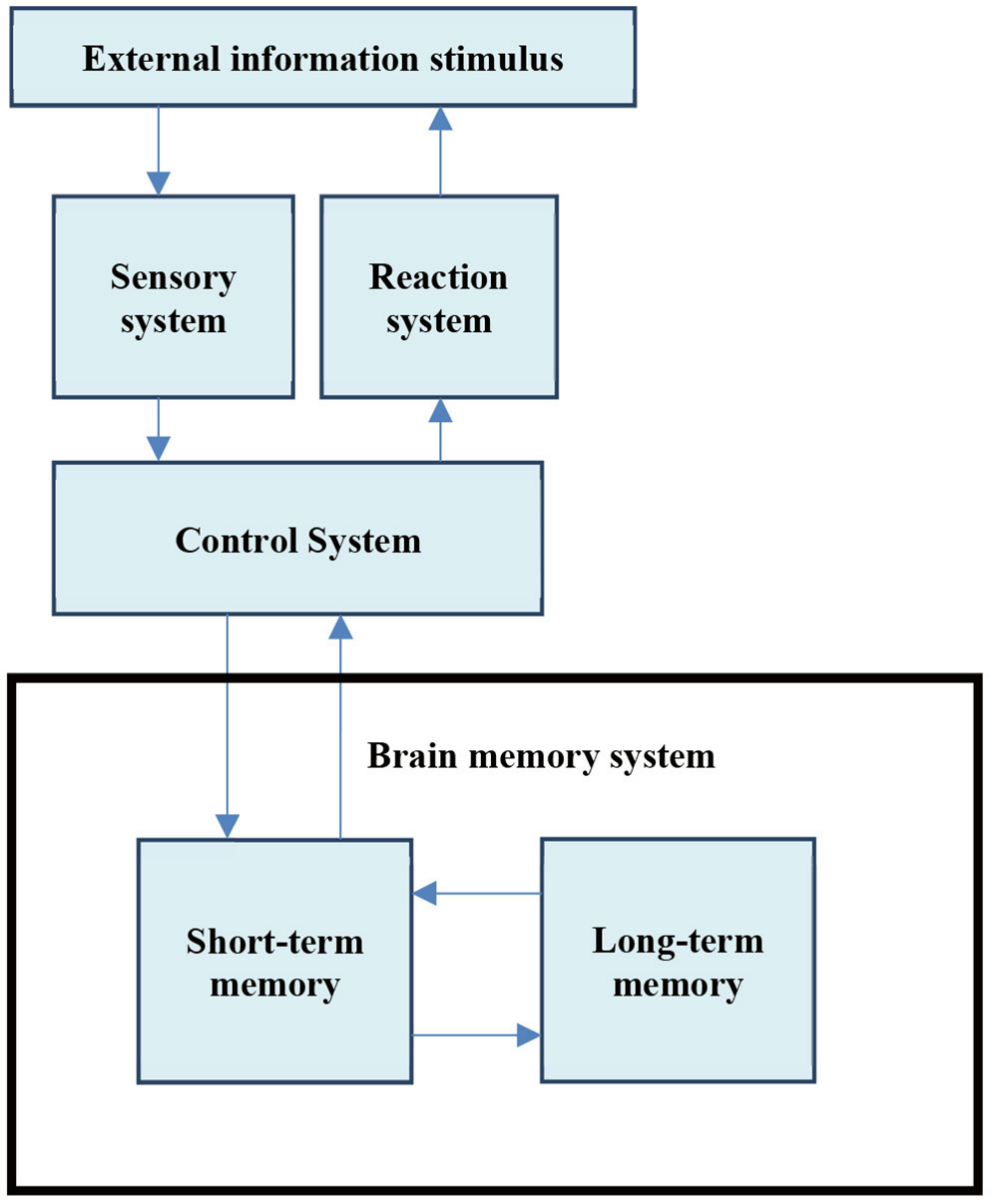
Information processing model.

Information processing psychology regards people's cognitive process as the process of inputting, coding, storing, extracting, and using information, advocates to study the structure and process of mental activities, and views these psychological processes as information processing processes (9). It presents the new direction and mode of psychological development and is widely used in teaching practice. The human information processing process model is shown in Figure 1.

As Figure 1 shows, the information is temporarily stored in the original sensory form in the sensory system, and enters the short-term and long-term memory through the control system for information storage. The information retained in the long-term memory is extracted into the short-term memory, and the current task is to solve the problem. Besides, human information processing also shows the influence of the latter processing stage on the former processing stage. Information processing is in chronological order and is bidirectional (10, 11).

**Figure 2:**
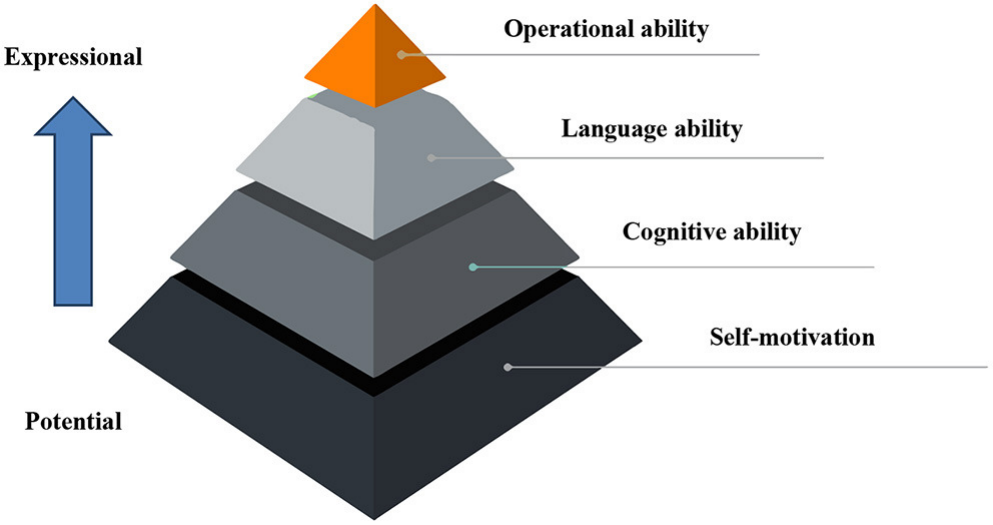
English communication ability system.

English communication ability is mainly determined by self-motivation, language ability, cognitive ability, and operational ability (12). The model system is shown in Figure 2.

**Figure 3:**
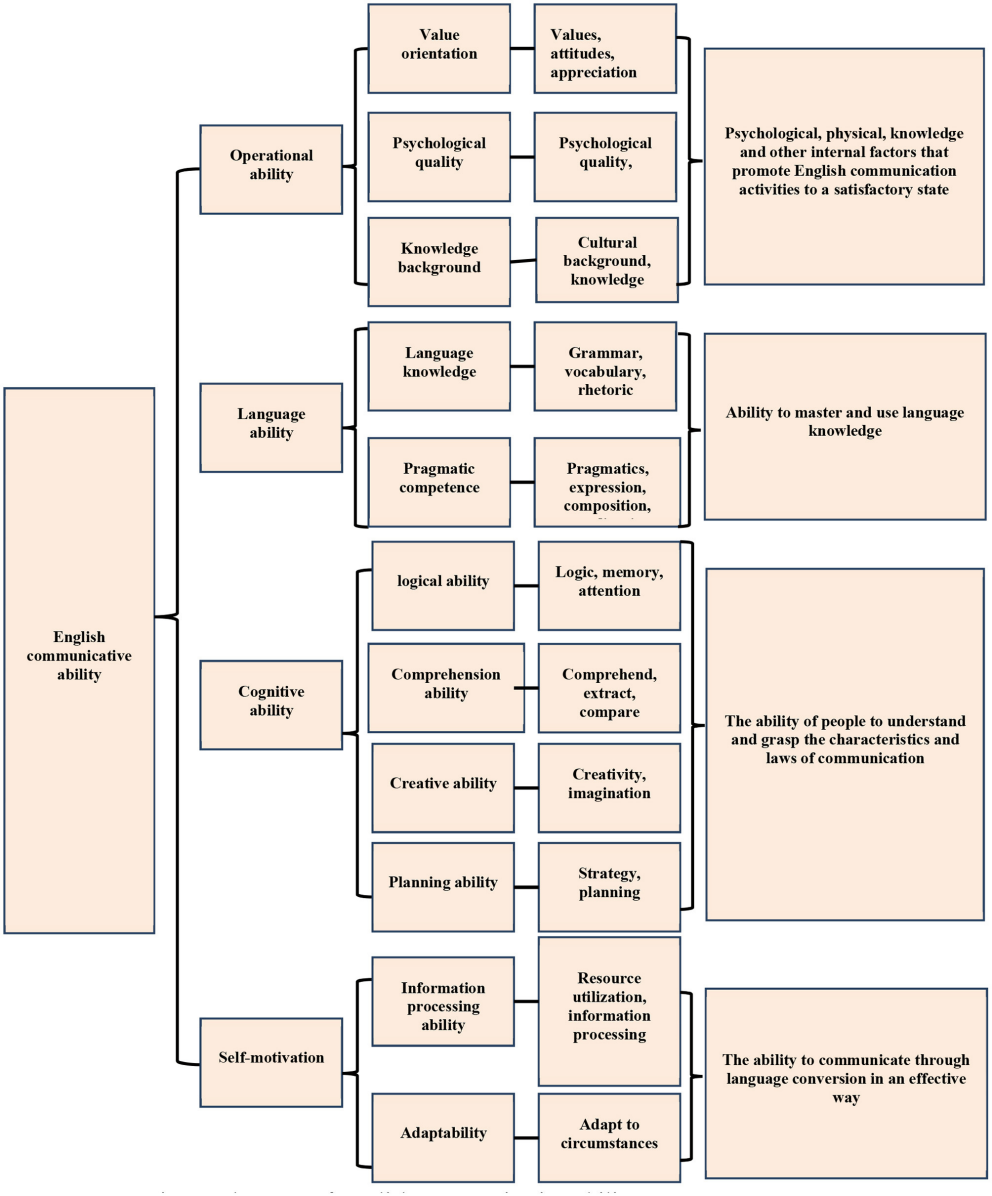
Constituent elements of English communication ability.

Figure 2 shows that the bottom of the pyramid is self-motivation, and the top is operational ability. The constituent elements are gradually from the internal to the external. The aspects of self-motivation are the psychological, physiological, knowledge, and other internal factors that promote English communication activities. Language ability is the ability to master and apply language knowledge to practice. Cognitive ability is people's ability to know about and pick up the characteristics and laws of communication. Operational ability is the ability to carry out language conversion and transmission in an effective way (13–15). The constituent elements of English communication ability are shown in Figure 3.

Figure 3 depicts the three secondary factors of self-motivation: value orientation, psychological quality, and knowledge base. Values, attitudes, and appreciation are all part of value orientation. Psychological quality is divided into three categories: personality, self-confidence, and psychological quality. The cultural and knowledge backgrounds are the knowledge backgrounds. Language competency includes both linguistic and pragmatic abilities. Language knowledge includes three tertiary elements: grammar, vocabulary, and rhetoric, and pragmatic competence includes four tertiary elements: pragmatics, expression, synthesis, and coordination. Cognitive ability includes the logical ability, understanding ability, creative ability, and planning ability. The logical ability has three levels: logic, memory, and attention. Understanding ability covers comprehension, extraction, and comparison. Creative ability involves creation and association. Planning ability includes strategy formulation and plan-making. Operational capability has information-capturing capability and reaction ability. Information ability includes resource utilization and information processing, and contingency capability is the reaction ability when a situation occurs (16–19).

### Training Methods

The main problem that students face in English communication is that they can't speak or dare not speak. The main reasons are that: they don't know the pronunciations of words, translation rules, and language points; they have little training time; they lack interest in English because of long-term mechanical reading, writing, and translation. Students' interest in English should be aroused in response to the problems. Their memory ability and response-ability should be trained based on cognitive psychology, achieving students' smooth communication in English.

**Figure 4:**
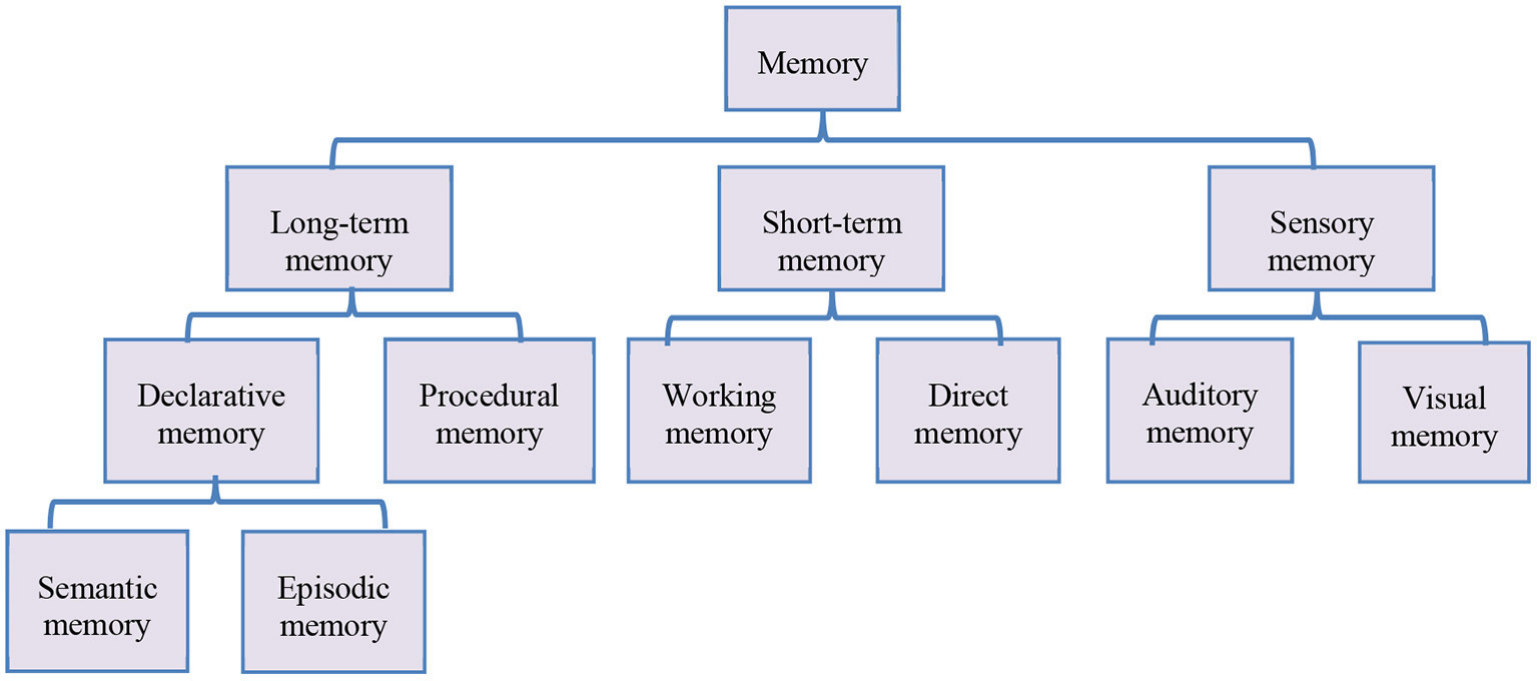
Memory classification.

Memory is divided into sensory, short-term, and long-term memories in cognitive psychology. Visual and auditory memory relate to the processing of visual and auditory impressions after they have lasted for a length of time. Direct memory and working memory are two types of short-term memory. The capacity of direct memory, which is the memory of unprocessed original information supplied to the brain, is restricted. Working memory is a type of memory in which the brain re-encodes information from direct memory, increasing the brain's capacity. Long-term memory is declarative memory and procedural memory. Declarative memory is the memory of relevant facts, which can be described and reported through language and subdivided into situational and semantic memory. Procedural memory is based on skilled actions, which cannot be described in language, and needs many attempts and exercises to be gradually obtained (20, 21). The structure of memory is shown in Figure 4.

**Figure 5:**
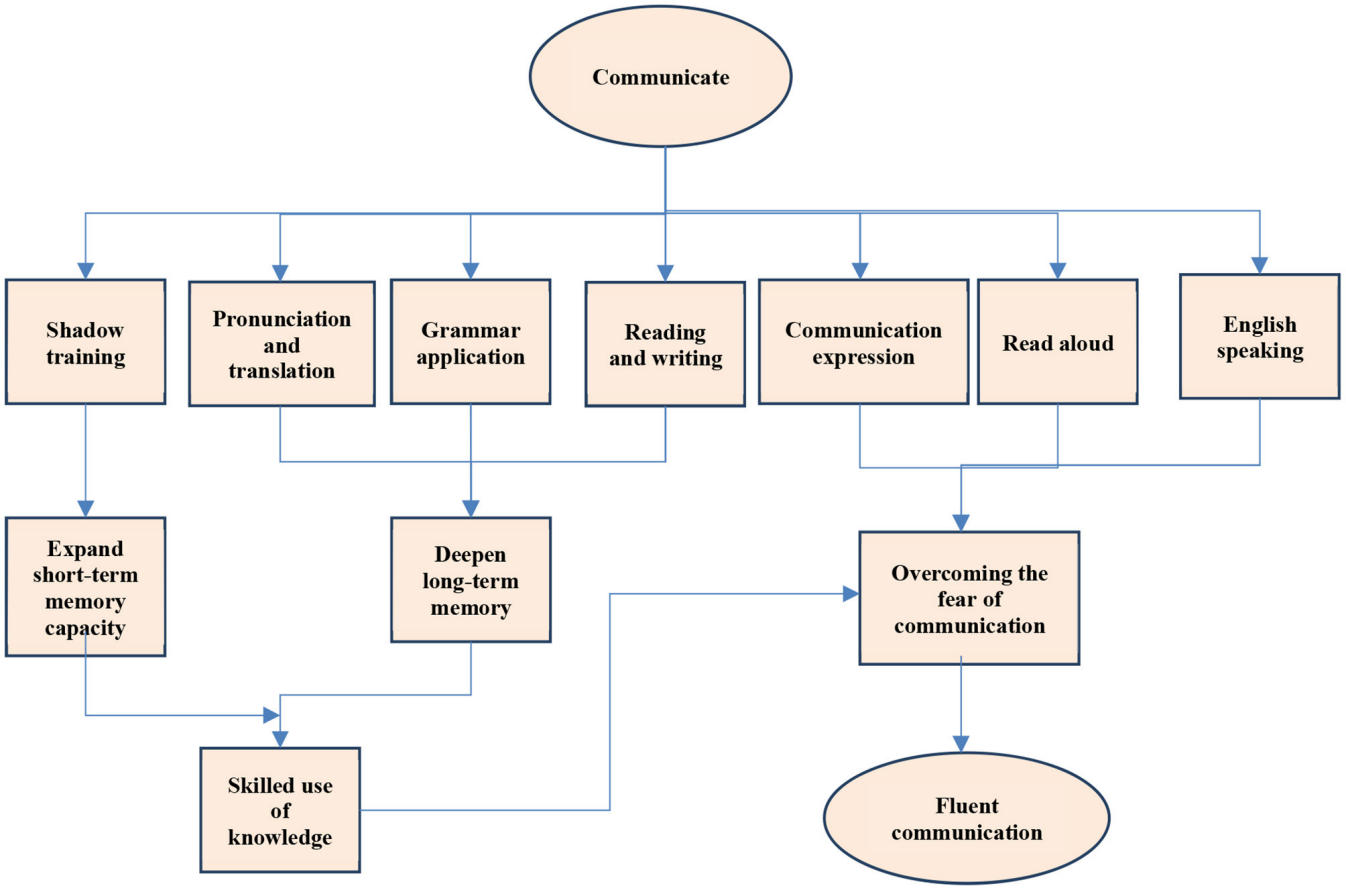
The model of English communication ability.

Combined with the causes of students' problems in English communication and the classification of memory, centralized training and practice are carried out from cognitive psychology. It mainly includes three aspects: expanding short-term memory capacity, deepening long-term memory, and overcoming communication fear. The model of improving English communication ability is shown in Figure 5.

The most important aspect of the memory system is short-term memory. There is no long-term memory without short-term memory. However, short-term memory capacity is limited, and an individual's memory range is likewise limited. As a result, the growth of short-term memory capacity is important for English communication fluency. Because English communication necessitates a large vocabulary and grammatical reserve as well as on-the-job adaptation, shadow training is the most effective training option (22). Shadow training, also known as primitive retelling exercise, is a way of practicing simultaneous interpreting abilities for interpreters, which enhances listening and speaking synchronization as well as short-term memory capacity. During shadow training, interpreters virtually synchronously follow the speech or pre-recorded recording in the same language. This method is very effective for memory and brain training. In the initial stage of training, students repeat the speech or recording to ensure the integrity and coherence of the content. After they are proficient, they can appropriately delay the speech or recording. The time difference can be kept at about half a sentence because listening, analysis, and output need to be completed quickly. This training method can significantly shorten the time required and strengthen the ability of brain division (23).

Long-term memory is divided into declarative memory and procedural memory. In the primary stage of English communication, it needs to know about the knowledge types, which often goes through three processes: “expounding the declarative knowledge, representing the knowledge, and figuring out solutions” (24). At this stage, learners always think about using words, phrases, and grammar correctly. Therefore, it is necessary to train how to memorize words, phrases, and grammar used in communication. The pronunciation, translation, terms, and grammar of words and their new references should be repeated every day, and review the previous ones until they can be spoken out naturally. Then, the review frequency can be appropriately reduced to ensure using them regularly. With the gradual formation of procedural knowledge, communicators gradually get rid of their dependence on declarative knowledge. Declarative knowledge representation is transformed into procedural knowledge representation. In the process of communication, there is no need to consciously search the vocabulary, phrases, and grammar in memory, forming a subconscious automatic response, making the communication more fluent and smooth. In deepening long-term memory, the degree of memory activation affects the speed and possibility of declarative information, and the frequency and recent degree of memory affect the degree of activation. Therefore, in-depth practice can effectively enhance the learners' memory ability and improve the speed of information extraction and retrieval, realizing effective English communication (25).

Learners' fear in English communication stems from their inability to skillfully apply knowledge into communication and people's ignorance of oral English communication, resulting in few opportunities for students to communicate in English. For the former, it can be effectively solved by expanding the capacity of short-term memory and deepening long-term memory. The latter still needs a lot of practice to integrate English communication into the communicators' learning and life, making them adapt to English environments quickly and improve their English communication skills.

## Results and Discussion

### Experimental Design

Sixty students from North China University of Water Resources and Electric Power are selected as the research subject. They are divided into classes A and B. Class A is defined as the experimental group and class B as the control group. Two oral tests with the same level but different contents are set for the pre-test and post-test. The test has four parts: reading comprehension, question answering, situational dialogue, and topic description. These four parts cover all aspects of English communication and expression. Passage reading examines the standard of pronunciation and intonation. Answering questions tests the students' ability to understand and respond to the passage. Situational dialogue shows coping ability and language ability. The topic description tells reaction ability and language ability. The scores of the four parts are 10 points for each. They are scored according to their pronunciation, intonation, expressions, the use of grammar, and the coherence of narration. The specific scoring standards are shown in Table 1.

**Table 1:** Scoring criteria for oral English test.

**Test items**	**Levels**	**Scores**	**Scoring standards**
Reading comprehension	Level 1	9–10	Correct Excellent pronunciation, natural intonation, fluent reading, and good rhythm
	Level 2	6–8	Basically correct pronunciation, natural intonation, and fluent reading
	Level 3	3–5	Having few mistakes in pronunciation and intonation, incoherent reading
	Level 4	0–2	Having many mistakes in pronunciation and intonation, incoherent reading
Question answering	Level 1	9–10	Clear expression, excellent pronunciation, natural intonation, and having no mistakes in grammar
	Level 2	6–8	Clear expression, correct pronunciation, natural intonation, and having few mistakes in grammar
	Level 3	3–5	Vague expression, having some mistakes in pronunciation, intonation, and grammar
	Level 4	0–2	Incorrect expression, having many mistakes in pronunciation, intonation, and grammar
Situational dialogue	Level 1	9–10	Clear expression, excellent pronunciation, natural intonation, and having no mistakes in pronunciation, intonation, and grammar
	Level 2	6–8	Basically clear expression, correct pronunciation, and natural intonation having few grammatical mistakes
	Level 3	3–5	Unclear expression, having many mistakes in pronunciation, intonation, and grammar
	Level 4	0–2	Just speaking out a few words related to the content, incorrect pronunciation
Topic description	Level 1	9–10	Excellent pronunciation, natural intonation, clear expression, no <10 sentences
	Level 2	6–8	Basically correct pronunciation, coherent narration, and clear expression, no <10 sentences, having few mistakes in pronunciation, intonation, and grammar
	Level 3	3–5	Basically correct pronunciation, coherent narration, no <10 sentences, having some mistakes in pronunciation, intonation, and grammar
	Level 4	0–2	Just speaking out a few words related to the content, incorrect pronunciation

The students in classes A and B take an oral English examination, with two college English instructors serving as examiners. After the test, the students of class A are trained for 2 months. The students of class B are only informed of the post-test time. The training of class A is conducted as follows: (1) each student spends 15 minutes to practice their oral English every day. The students are divided into five groups, with 6 in each group. A daily communication topic is selected in the group every day. The group members communicate independently in English and are supervised by the team leader. (2) Each student reads English articles aloud for 15 minutes every day. The group members help each other correct their mistakes in time. (3) Each student prepares 3 min of English speech every day. The topic is optional. The activity is held in the group and supervised by the team leader. (4) Each student writes an English essay every week with about 500 words on any topic. Most of them are discussed topics in daily communication, which can exercise students' language organization ability. (5) Read 1,500 words of English materials every week. Through reading English materials, students can accumulate materials, have more topics to communicate in oral communication and understand more English-related cultural and language expression structures. (6) Each student memorizes 50 words or phrases every day. It is not required to spell out, but to recall pronunciation and English-Chinese translation. The team leader is also responsible for the check task. The team leader is needed to record the video in the training process every day. After the 2-month training, classes A and B students are given a post-test. The test is consistent with the pre-test. Finally, the pre-test and post-test scores of the students in the two classes are compared.

### Experimental Result

**Figure 6:**
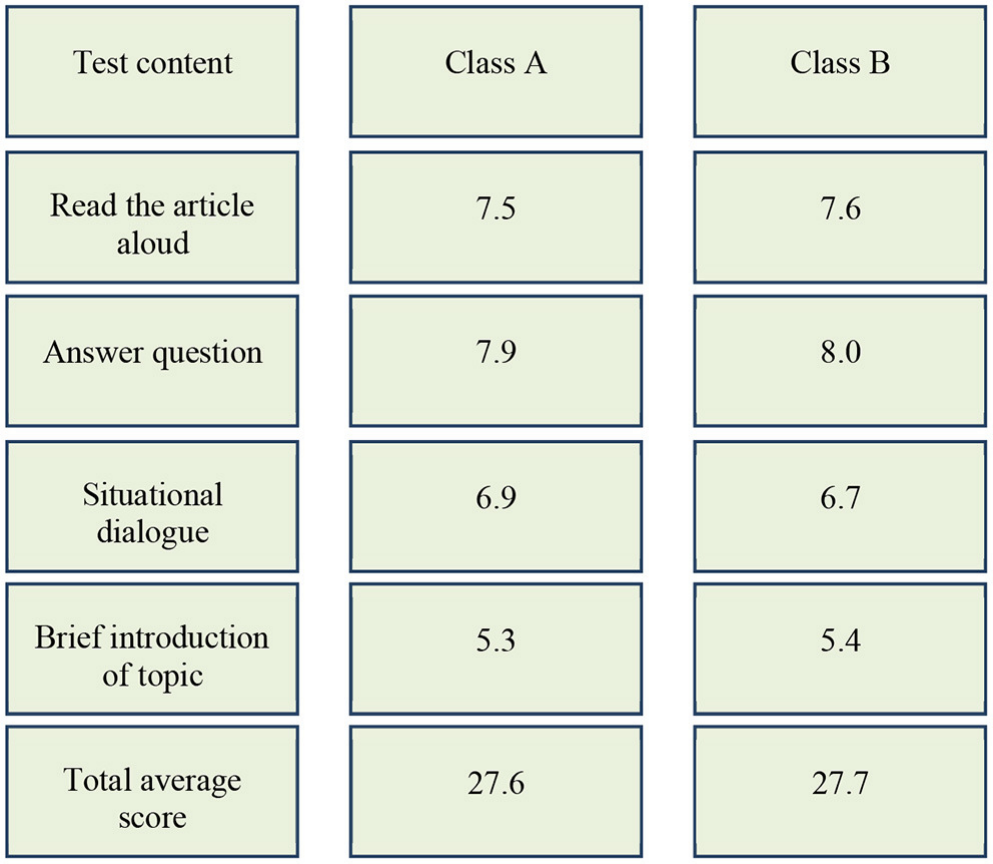
Comparison of pre-test scores of students in classes A and B.

In the pre-test, the test results of students in classes A and B are shown in Figure 6.

In the pre-test, the oral test scores of classes A and B are almost the same, and the average score of class B is slightly higher than that of class A by 0.1.

**Figure 7:**
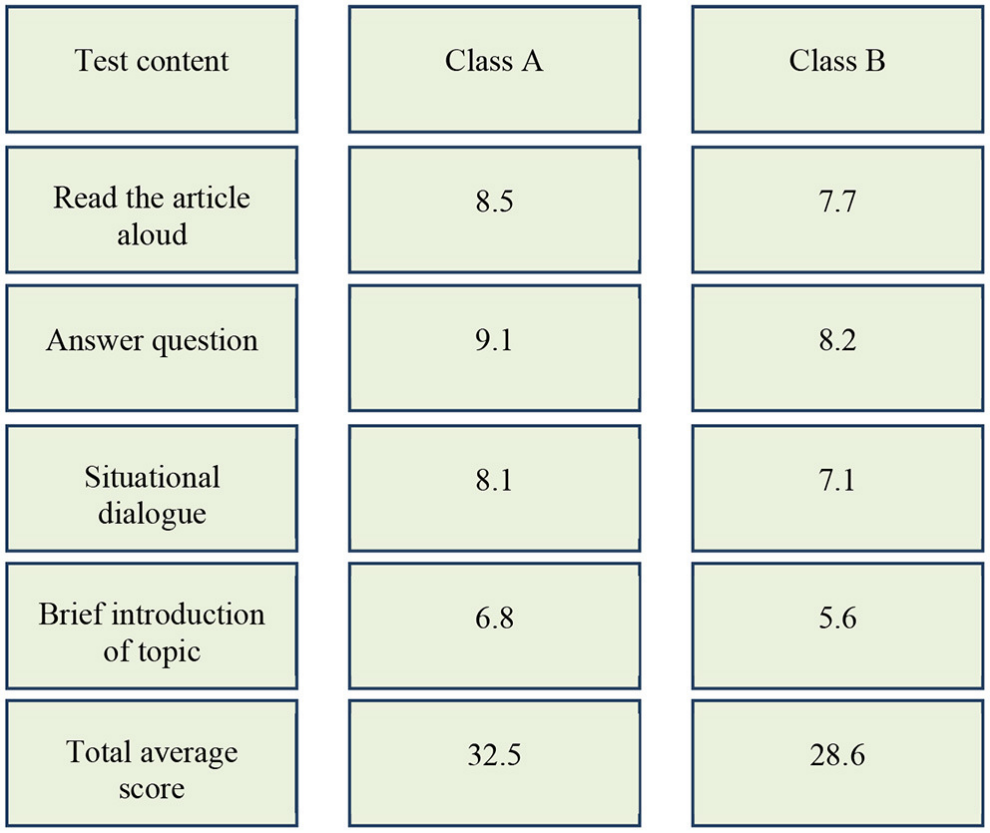
Comparison of post-test scores of students in classes A and B.

After two-month oral communication training, the post-test results are shown in Figure 7.

**Figure 8:**
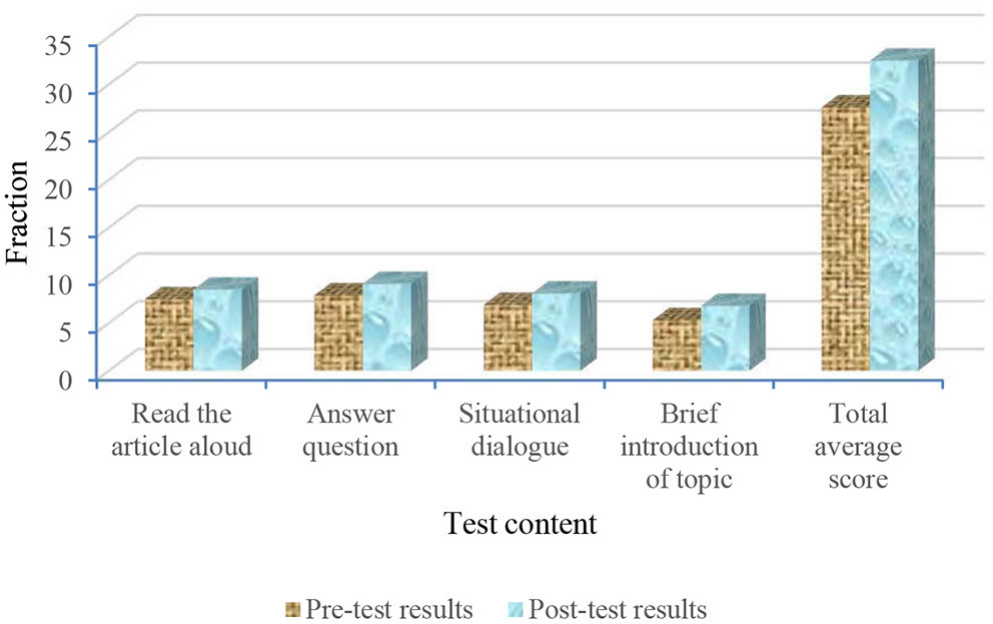
Comparison of pre-test and post-test scores of students in class A.

**Figure 9:**
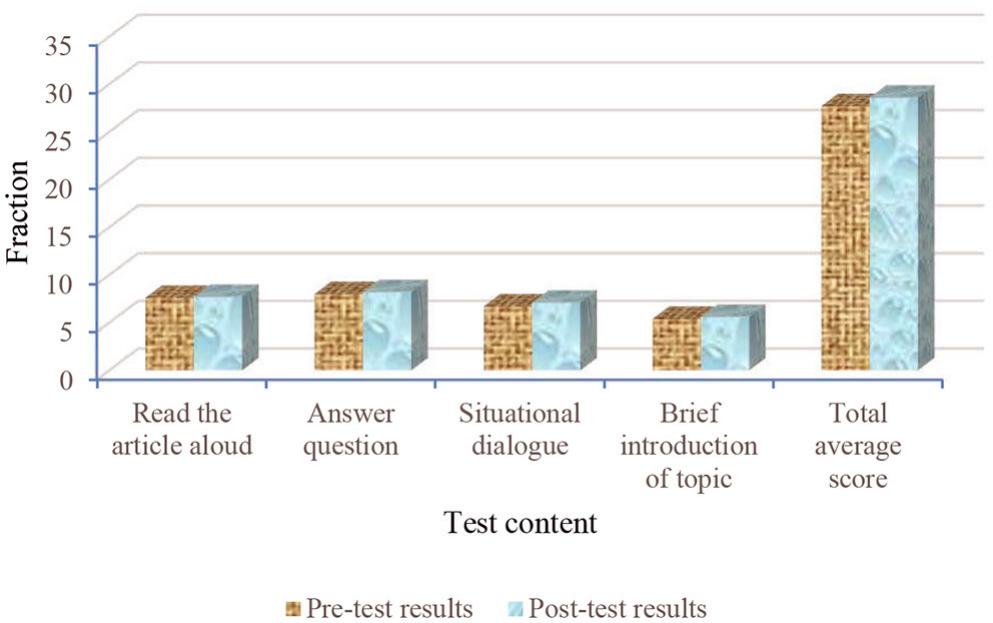
Comparison of pre-test and post-test scores of students in class B.

Figure 7 shows that the total average score of class A in the experimental group is 3.9 points higher than that of class B in the control group, although the oral communication scores of classes A and B improve after two months' training. The improvement ranges of classes A and B are shown in Figures 8, 9, respectively.

Figure 8 shows that the scores of the students in class A are significantly improved. The improvement ranges of the students' scores in classes A and B are shown, respectively. The calculation equation is as follows:


$$\begin{array}{l} X\;=\;\frac{a_{2}-a_{1}}{a_{1}}\;\times 100\;\% \end{array}$$

In Equation (1), *X* is the improved range of pre-test and post-test scores, *a*_2_ indicates the scores of the post-test, and *a*_1_ indicates the score of the pre-test. Through calculation, the reading comprehension scores in class A increased by 13.33%, answering questions by 15.19%, situational dialogue by 17.39%, topic description by 28.3%, and the total average score of the whole class increased by 17.75%. Figure 9 shows that the scores of reading comprehension in class B increase by 1.32%, answering questions by 2.5%, situational dialogue by 5.97%, topic description by 3.7%, and the total average score of the whole class increases by 3.25%. Compared with class B, the scores of the students in class A are greatly improved, which shows that the standard degree of pronunciation and intonation, understanding ability, response-ability, coping ability, and language ability of the students in class A is improved in English communication training. This proves that the model and strategy based on cognitive psychology are scientific and feasible.

### Analysis of Neural Network Algorithm

We conduct a comparison simulation test based on a range of neural network algorithms in order to more clearly identify the performance of neural network algorithms in the model. We specify uniform settings for each variable in the test training to ensure that the test outcomes are identical. We set the hyper parameters i.e., the number of input and output nodes, number of hidden layers and hidden nodes in each layer and the maximum training iterations. We adjust the weights and migration variables according to the optimization function to guarantee that the detection simulation structure has the best solution. For the performance comparison we considered the classifiers including: K-Nearest Neighbor-KNN (26), Random Forest-RF (27), and Support-Vector-Machine-SVM (27–29). The performance comparison of different classifiers using the accuracy (ACC), specificity (SP), sensitivity (SN) and Mathew's Correlation Coefficient (MCC) as shown in Table 2.

**Table 2:** Experimental comparison results.

**Model**	**SN (%)**	**SP (%)**	**ACC (%)**	**MCC**
Proposed model	94.22	90.32	93.54	0.89
SVM	90.14	87.29	89.40	0.783
KNN	86.46	80.13	85.65	0.68
Random Forest	87.14	84.47	85.53	0.67

Table 2 shows that the DNN model attained the best accuracies, 93.54%, compared to other classifiers. DNN model achieved the highest performance metrics, for example, the DNN model achieved a sensitivity of 93.22%, specificity of 90.32%, and MCC 0.89. Furthermore, the findings reveal that the DNN model enhanced accuracy by an average of 10.41%. As a result, the DNN was chosen as the final classifier in the proposed model.

## Conclusion

To increase pupils' English communication abilities, a paradigm based on cognitive psychology is used. The tactics for improving students' English communication abilities are suggested and applied on this premise. First, an in-depth examination of cognitive psychology is carried out. Second, the issues and factors impacting students' English communication skills are investigated. Finally, techniques for improving English communication skills are developed. The research subjects are 60 students from North China University of Water Resources and Electric Power. They are separated into two groups: A and B. The experimental group is called Class A, while the control group is called Class B. After the test results before and after training are compared, it is found that the scores of the students in reading comprehension, answering questions and situational dialogue, and topic description in class A increase by 13.33, 15.19%, and 17.39, and 28.3%, and the total average score of the whole class increases by 17.75%. The improvement rate of class B in the two tests is minimal.

Moreover, it is proved that the model and strategy based on cognitive psychology are scientific and feasible. Due to the small size of samples in this experiment, the errors in the investigation are considerable. The proposed model exhibited promising results however, the model needs high-performance computing to deliver satisfactory findings promptly because of its great computational complexity. In the future, the data accuracy will be improved. The study has a reference for improving students' English communication ability.

## Data Availability Statement

The original contributions presented in the study are included in the article/supplementary material, further inquiries can be directed to the corresponding author/s.

## Author Contributions

YW and LZ contributed to conception and design of the study. YW organized the database and wrote the first draft of the manuscript. LZ performed the statistical analysis. All authors contributed to manuscript revision, read, and approved the submitted version.

## Funding

This study was supported by Educational Reform Project in School of Foreign Studies of NCWU: Research on the Construction of College English Courses based on the Improvement of Language Literacy and the Cultivation of International Communication Skills.

## Conflict of Interest

The authors declare that the research was conducted in the absence of any commercial or financial relationships that could be construed as a potential conflict of interest.

## Publisher's Note

All claims expressed in this article are solely those of the authors and do not necessarily represent those of their affiliated organizations, or those of the publisher, the editors and the reviewers. Any product that may be evaluated in this article, or claim that may be made by its manufacturer, is not guaranteed or endorsed by the publisher.
